# Pancreatic intraductal tubulopapillary neoplasm progression requiring completion pancreatectomy: A case report and literature review

**DOI:** 10.1016/j.ijscr.2020.10.037

**Published:** 2020-10-12

**Authors:** Joshua T. Cohen, Marsela Hyska-Campbell, Abigail L. Alexander, Elizabeth Yiru Wu, Pranith N. Perera, Rachel E. Beard

**Affiliations:** a Department of Surgery, Rhode Island Hospital, Warren Alpert Medical School of Brown University, Providence, RI, United States; b Department of Radiology, Rhode Island Hospital, Warren Alpert Medical School of Brown University, Providence, RI, United States; c Department of Pathology, Rhode Island Hospital, Warren Alpert Medical School of Brown University, Providence, RI, United States; d Department of Gastroenterology, Rhode Island Hospital, Warren Alpert Medical School of Brown University, Providence, RI, United States

**Keywords:** Intraductal tubulopapillary neoplasm, Pancreatic tumor, Case report

## Abstract

**Introduction:**

Intraductal tubulopapillary neoplasm (ITPN) is a recently described rare tumor of the pancreas. Diagnostic approach and treatment are based on relatively few cases.

**Presentation of case:**

Here we report a case of a 68-year-old male presenting with an ampullary adenoma with high grade dysplasia who underwent pancreaticoduodenectomy and was incidentally found to have an ITPN at the pancreatic resection margin with areas of microinvasion throughout the resected specimen. He went on to rapidly develop an invasive adenocarcinoma arising in association with recurrent ITPN in the remnant pancreas requiring a completion total pancreatectomy.

**Discussion:**

Patients with ITPN present with non-specific symptoms and diagnosis can be challenging. Radiographic evaluation will reveal tumor ingrowth into the main pancreatic duct and distal duct dilatation without upstream dilation or mucinous engorgement. ITPNs are treated with formal resection given that determination of an invasive component can be difficult and the risk of malignant transformation. Following resection, recurrences are infrequent and 5-year survival is over 70 % even with microinvasion.

**Conclusions:**

ITPNs can follow a variable clinical course but hold the potential for malignant transformation. When ITPN is incidentally found at a pancreatic resection margin, we recommend completion resection due to the risk of local recurrence.

## Highlights

• Intraductal tubulopapillary neoplasm (ITPN) is a rare pancreatic tumor.
• Treatment of ITPN requires a formal pancreatic resection.
• ITPN at a pancreatic resection margin holds the potential for rapid recurrence.

## 1 Introduction

ITPN is a relatively recently described, rare, pre-malignant intraductal epithelial tumor of the pancreas distinct from intraductal papillary mucinous neoplasm [[1], [2], [3]]. ITPNs are most frequently found in the head of the pancreas and cause varying degrees of ductal dilatation [2,3]. On microscopic examination, these tumors lack intracellular mucin and predominantly demonstrate a tubular growth pattern, with true papillary formation in the minority of cases [2]. Magnetic resonance cholangiopancreatography (MRCP) and triple-phase CT scan can demonstrate the cork-of-wine-bottle sign or the two-tone duct sign, in which tumor ingrowth is observed in the lumen of pancreatic duct, similar to a cork in the neck of a wine bottle [4]. These lesions hold the potential for malignant degeneration with larger tumor size and higher Ki-67 index being associated with invasive ITPNs [3,5]. Herein we report a case of ITPN with microinvasion discovered in a pancreaticoduodenectomy specimen resected for ampullary adenoma with high grade dysplasia. The ITPN recurred in the remnant pancreas in association with invasive adenocarcinoma requiring a completion pancreatectomy which to our knowledge has not previously been reported.

## 2 Presentation of the case

**Fig. 1:**
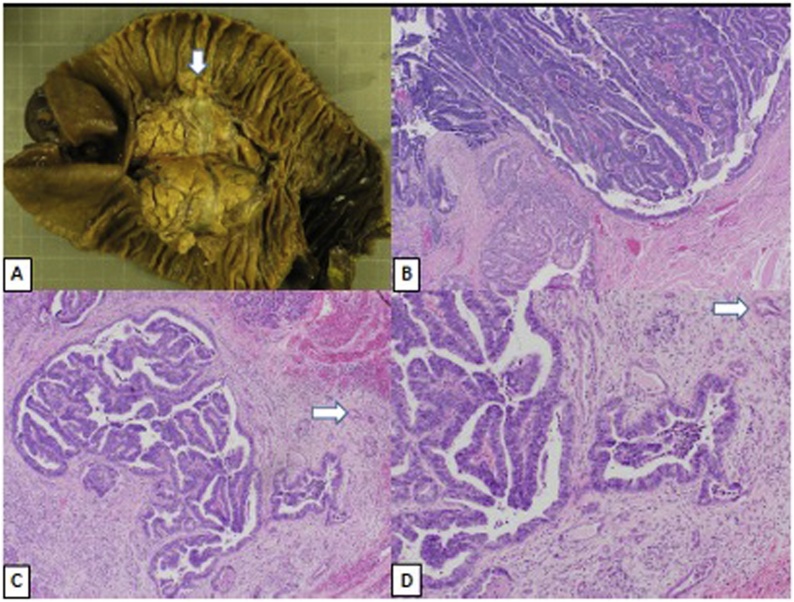
(A) Coronal T2 weighted MR abdomen examination showing a pancreatic head ductal lesion (red arrow). (B) Axial T2 weighted MR and (C) axial contrast enhanced CT abdomen showing normal pancreatic body and tail.

A 68-year-old male initially presented with recurrent pancreatitis, initially attributed to cholelithiasis for which he underwent a laparoscopic cholecystectomy. Postoperatively, he continued to have episodes of pancreatitis which prompted an MRCP that demonstrated dilation of the common bile duct and pancreatic duct to 8 mm and 4 mm respectively (Fig. 1A) with a normal pancreatic body and tail (Fig. 1B). He then underwent an endoscopic ultrasound (EUS) which demonstrated a 16 × 11 mm ampullary mass. Biopsies demonstrated moderately differentiated adenocarcinoma, at least intramucosal with areas highly suspicious for invasive disease. CT scan of his chest, abdomen, and pelvis revealed no additional pancreatic lesions (Fig. 1C) or evidence of metastasis.

**Fig. 2:**
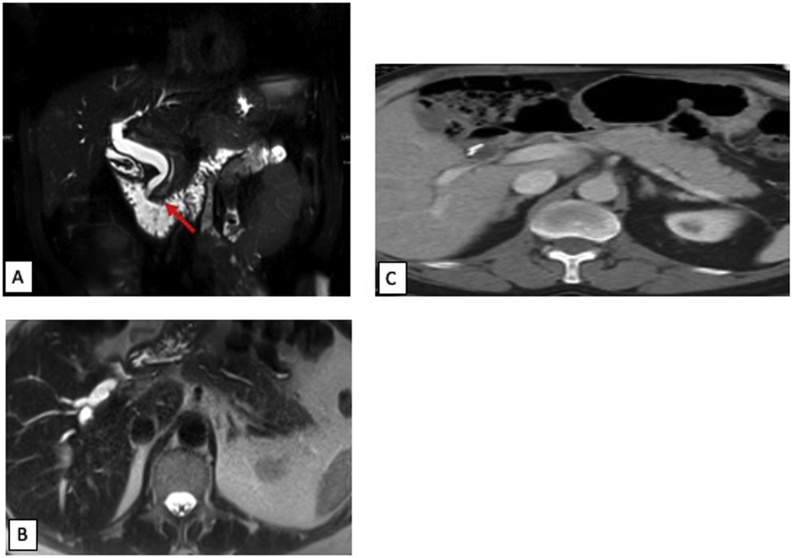
(A) Partial pancreatoduodenectomy specimen; white arrow denotes ampullary adenoma. (B) Ampullary adenoma with high-grade dysplasia; peri-ampullary glands at left lower. Intraductal tubulopapillary neoplasm with microinvasion (white arrow) at (C) low and (D) high power.

Following a negative diagnostic laparoscopy, a pancreaticoduodenectomy was performed by a faculty surgeon (Fig. 2A). Frozen pathology sections from the pancreatic and bile duct margins were examined and were negative for carcinoma or high-grade dysplasia. His postoperative course was complicated by pneumonia requiring antibiotics and a chyle leak which resolved with TPN and a low-fat diet. Final pathology demonstrated two lesions. The first was a 17 × 11 × 8 mm ampullary adenoma with predominantly intestinal morphology and high-grade dysplasia but no evidence of invasion (Fig. 2B). The second lesion, not grossly evident, was characterized by an intraductal proliferation with mixed tubular and papillary architecture, comprised of non-mucinous epithelial cells with variable cytologic atypia, consistent with an intraductal tubulopapillary neoplasm (ITPN; Fig. 2C). Multifocal microinvasion (<2 mm in greatest contiguous dimension) was associated with the ITPN (Fig. 2C and D) but did not extend to the pancreatic neck margin. The pancreatic neck margin was, however, focally positive for ITPN without high-grade dysplasia. There was no invasive carcinoma at the resection margin. Eight lymph nodes examined and were negative for carcinoma. Given the rarity of this disease, sections were forwarded to Memorial Sloan Kettering Cancer Center where the diagnosis was confirmed.

**Fig. 3:**
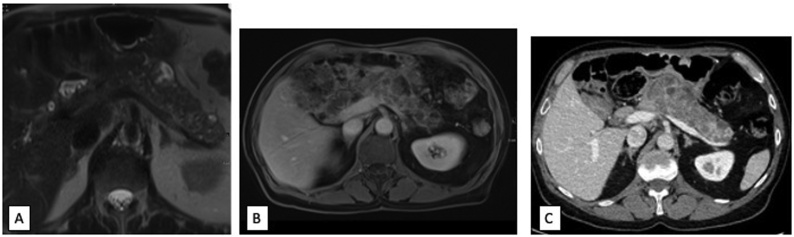
(A) Axial T2 weighted MR abdomen showing enlargement of the pancreatic body and tail with multiple new cystic lesions 8 months post-operative from the index operation. (B) Axial post-contrast MR abdomen at 11 months post-operative from the index operation and (C) axial contrast enhanced CT abdomen showing heterogeneous contrast enhancement of the body and tail at 15 months post-operative from the index operation.

Pathology findings were discussed at multidisciplinary tumor board and with the patient. Given that there was only a single small (<2 mm) focus of ITPN without high-grade dysplasia the decision was made to continue close monitoring. Surveillance CT scan three months following his operation demonstrated a normal remnant pancreas. Eight months after his index operation, however, an MRCP was performed that demonstrated multicystic appearance throughout the gland with a dominant pancreatic body cystic lesion measuring 2.6 cm with enhancing mural nodularity, felt to represent either pancreatitis with developing pseudocysts or possibly evolving malignancy (Fig. 3A). Given the background pancreatitis, a multidisciplinary tumor board felt the most likely diagnosis of the cystic lesions was a pseudocyst and the decision was made to continue close monitoring with serial imaging and to defer cytologic examination of the cyst fluid.

Three months later, he underwent repeat MRCP which demonstrated progression of the previously noted multicystic appearance of the remnant pancreas with diffusion restriction on delayed imaging (Fig. 3B). Given these findings, EUS was performed which demonstrated diffuse parenchymal abnormalities including echogenicity and heterogeneity of the gland with a dominant 2.9 cm cyst in the body, with internal debris and without clear communication with the pancreatic duct. Cyst fluid cytology was consistent with ITPN with high grade dysplasia, suspicious for adenocarcinoma. Cyst fluid analysis demonstrated a CEA of 1261 ng/mL, amylase 262 U/L and PancraGEN analysis classified the cyst as statistically high risk based on low clonality KRAS point mutation and multiple loss of heterozygosity (LOH) mutations. Preoperative staging CT scan demonstrated known heterogeneous contrast enhancement of the body and tail of the pancreas (Fig. 3C) without evidence of metastatic disease.

**Fig. 4:**
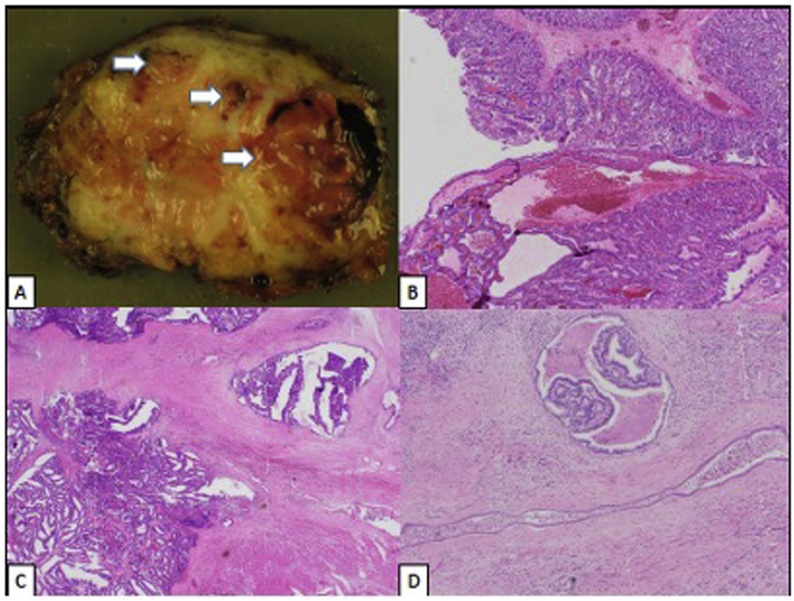
(A) Cut section of completion pancreatectomy with intraductal growth of tumor (white arrows). (B) Confluent growth of adenocarcinoma invading transmurally into the lumen of the small intestine (normal small intestine in upper half), and (C) through muscularis propria of small intestinal wall with stromal reaction (residual muscularis propria in lower right). (D) The background pancreas demonstrated diffuse involvement of the duct system by ITPN.

Fifteen months after his index operation the patient, was taken back to the operative room for a completion total pancreatectomy and splenectomy. He had an uncomplicated hospital course and was discharged home on post-operative day 6 when a stable insulin regimen was determined. The completion pancreatectomy and fragment of small intestine underwent pathologic examination. Opening of the small intestine demonstrated detached friable tumor within the lumen. Serial sectioning of the specimen demonstrated diffuse heterogeneity of the remnant gland with evident distension of residual duct spaces with tumor (Fig. 4A). Histologic examination of the junction between small intestine and pancreas demonstrated confluent growth of a tubulopapillary proliferation with associated stromal reaction consistent with invasive adenocarcinoma, transmurally involving the small intestinal wall (Fig. 4B and C). The background pancreas was remarkable for diffuse involvement of the duct system by ITPN (Fig. 4D). The total size of the invasive component spanned the entirety of the remnant gland (11.5 cm), and measured 0.5 cm from the small intestinal resection margins. There was no carcinoma in the spleen. Twenty-five lymph nodes examined and were negative for carcinoma. He is now eight months out from his second operation with no evidence of disease recurrence after completing adjuvant gemcitabine and capecitabine. This case has been reported in line with the SCARE 2018 criteria [6].

## 3 Discussion

ITPN is a recently described rare entity that remains challenging to identify preoperatively [[2], [3], [4], [5]]. Here we report a case of ITPN found incidentally at the resection margin of a pancreaticoduodenectomy performed for a distinct ampullary adenoma that went on to rapidly progress to an invasive adenocarcinoma. To our knowledge, this is the first report of a case of incidentally identified ITPN at a resection margin.

Due to the limited number of cases, the diagnosis of ITPN can be challenging. Forty-five to 59 % of ITPNs are located in the pancreatic head which comprises the most common location [2,3,5,7]. Abdominal pain is the most common presenting symptom, although patients will also present with jaundice, abdominal fullness, diarrhea, worsening diabetes mellitus, pancreatitis, and excessive thirst. Between 26.7 % and 54.5 % of patients will be asymptomatic at the time of presentation [2,5,7].

Motosugi et al. identified the 2-tone duct sign on cross-sectional imaging and EUS and the cork-of-wine bottle sign on cholangiopancreatography as associated with ITPN in a study of 10 biopsy proven cases [4]. These signs are characterized by tumor ingrowth filling the main pancreatic duct comprising one tone and pancreatic fluid representing the other tone. On MRCP and endoscopic retrograde cholangiopancreatography (ERCP), the tumor may present as a filling defect that appears similar to a cork sitting in the mouth of a wine bottle. Distal pancreatic duct dilation was present in all cases of ITPN involving the main pancreatic duct while downstream duct dilatation is not observed as these tumors do not produce a large amount of mucin, in contrast to intraductal papillary mucinous neoplasm (IPMNs).

On pathologic examination, the majority of ITPNs are predominantly solid or polypoid lesions within the duct system; however, tumors may appear cystic in nature that demonstrate intraluminal growth without intraluminal mucin secretion [2]. Microscopically, they exhibit tubulopapillary growth with cuboidal to columnar cells and no or rare foci of cytoplasmic mucin. Tumors appear as large cribriform structures with surrounding fibrosis and will have foci of necrosis in the majority of cases [2,3]. Isolated areas of true papilla formation are seen in only 36 % of patients [2]. Due to the expansile nature of the tumor nodules, and the frequent lack of a normal layer of ductal epithelium to confirm intraductal growth, evaluation of invasion can be challenging, but can be identified as foci of tumor cells or clusters with surrounding desmoplastic reaction [2]. IPMNs and Pan-INs are distinguished morphologically from ITPNs by the presence of mucinous epithelium; immunohistochemically, ITPNs are characterized by negative MUC2 and MUC5AC reactivity, and absence of KRAS mutations [2,3,5].

Given the challenging nature of determining an invasive component and the risk of malignant degeneration, ITPNs are treated with formal pancreatic resection. Consistent with patterns of tumor location, the most common resection performed is a pancreaticoduodenectomy followed by a distal pancreatectomy and total pancreatectomy [2,3,5]. In a multi-institutional series of 33 cases, Basturk et al. reported four patients had local recurrence and two patients had liver metastasis at a median follow up of 61.5 months. Five-year survival was 77 % in patients without an invasive component and 71 % in patients with invasion, which was not significantly different [2].

## 4 Conclusion

ITPN remains a rare entity, and as such the diagnostic approach and treatment recommendations are based on a limited number of cases. Resection of ITPN with an invasive component confers a more optimistic long-term prognosis when compared with pancreatic adenocarcinoma. Although future studies are necessary, based on our clinical experience, we would advocate for completion total pancreatectomy when ITPN with microinvasion is present at the resection margin, regardless of the degree of dysplasia that is present based on the rapid progression in this case.

## Declaration of Competing Interest

The authors report no declarations of interest.

## Funding

We have no funding sources to declare.

## Ethical approval

This is an exempted study per the Lifespan Institutional Review Board.

## Consent

Written informed consent was obtained from the patient for publication of this case report and accompanying images. A copy of the written consent is available for review by the Editor-in-Chief of this journal on request.

## Author contribution

JTC: Study concept, data collection, writing the paper.

MHC: Data collection, writing the paper.

ALA: Data collection, writing the paper.

EYW: Data collection, writing the paper.

PNP: Study concept, writing the paper.

REB: Study concept, data collection, writing the paper.

## Guarantor

Rachel E. Beard.

## Provenance and peer review

Not commissioned, externally peer-reviewed.
